# Analysis of proteome and post-translational modifications of 2-hydroxyisobutyrylation reveals the glycolysis pathway in oral adenoid cystic carcinoma

**DOI:** 10.1186/s12957-023-03155-x

**Published:** 2023-09-23

**Authors:** Sining Chen, Dandan Li, Zhipeng Zeng, Wei Zhang, Hongliang Xie, Jianming Tang, Shengyou Liao, Wanxia Cai, Fanna Liu, Donge Tang, Yong Dai

**Affiliations:** 1 Clinical Medical Research Center, The Second Clinical Medical College of Jinan University (Shenzhen People’s Hospital), Jinan University, Shenzhen, 518020 Guangdong China; 2 Nephrology Department, The First Affiliated Hospital of Jinan University, Jinan University, Guangzhou, 510632 China; 3 Experimental Center, Shenzhen Pingle Orthopedic Hospital (Shenzhen Pingshan Traditional Chinese Medicine Hospital), Shenzhen, Guangdong 518118 China; 4 Department of Oral and Maxillofacial Surgery, Stomatological Medical Center, The Second Clinical Medical College of Jinan University (Shenzhen People’s Hospital), Shenzhen, 518020 Guangdong China; 5 Comprehensive health Industry Research Center, Taizhou Research Institute, Southern University of Science and Technology, Taizhou, 318000 China; 6 Department of Organ Transplantation, No.924 Hospital of PLA Joint Logistic Support Force, Medical quality specialty of the Joint Logistic Support Force, Guilin, 541002 China; 7 The first affiliated hospital, School of Medicine, Anhui University of Science and Technology, Huainan, Anhui 232001 China

**Keywords:** Glycolysis pathway, Oral adenoid cystic carcinoma, Lysine 2-hydroxyisobutyrylation, Proteomics, Therapeutics

## Abstract

**Purpose:**

Oral adenoid cystic carcinoma (OACC) has high rates of both local–regional recurrence and distant metastasis. The objective of this study is to investigate the impact of Khib on OACC and its potential as a targeted therapeutic intervention.

**Experimental design:**

We investigated the DEPs (differentially expressed proteins) and DHMPs between OACC-T and OACC-N using LC–MS/MS-based quantitative proteomics and using several bioinformatics methods, including GO enrichment analysis, KEGG pathway analysis, subcellular localization prediction, MEA (motif enrichment analysis), and PPI (protein–protein interaction networks) to illustrate how Khib modification interfere with OACC evolution.

**Results:**

Compared OACC-tumor samples (OACC-T) with the adjacent normal samples (OACC-N), there were 3243 of the DEPs and 2011 Khib sites were identified on 764 proteins (DHMPs). DEPs and DHMPs were strongly associated to glycolysis pathway. GAPDH of K254, ENO of K228, and PGK1 of K323 were modified by Khib in OACC-T. Khib may increase the catalytic efficiency to promote glycolysis pathway and favor OACC progression.

**Conclusions and clinical relevance:**

Khib may play a significant role in the mechanism of OACC progression by influencing the enzyme activity of the glycolysis pathway. These findings may provide new therapeutic options of OACC.

**Supplementary Information:**

The online version contains supplementary material available at 10.1186/s12957-023-03155-x.

## Statement of clinical relevance

The standard treatment options for OACC in clinical practice involve surgical intervention and radiotherapy. However, these interventions have a significant negative impact on patient’s quality of life, including changes in saliva volume, chewing ability, facial appearance, and verbal expression. To date, there has been a lack of standardization in drug treatments for OACC, and the progress in targeted treatments has been sluggish. The objective of this study was to investigate the impact of lysine 2-hydroxyisobutyrylation modification (Khib) on OACC and its potential as a targeted therapeutic intervention.

## Introduction

In February 2022, the China National Cancer Center indicated that the incidence of oral cancer in 2016 was 3.78 cases per 100,000 individuals annually [1]. Oral adenoid cystic carcinoma (OACC), accounting for less than 2% of malignant head and neck tumors (3–4.5 cases per million annually worldwide), is a rare tumor of oral cancer [2]. Due to its rarity, limited research has been conducted on the pathogenesis and treatment of OACC. OACC is distinguished by a significant occurrence of local–regional recurrence and distant metastasis. The survival rate of OACC within a span of 15 to 20 years is approximately 23 to 40% [3, 4]. Recurrence rates within the 5-to-10-year range vary from 30 to 75% [5]. To date, drug treatment for OACC have not been standardized and the progress on targeted treatment has been sluggish. The standard treatment options for OACC in clinical practice involve surgical intervention and radiotherapy [6, 7]. However, these interventions have a significant negative impact on patient’s quality of life, including changes in saliva volume, chewing ability, facial appearance, and verbal expression. Consequently, it is crucial to explore the pathogenesis of OACC and identify new targeted treatments.

Human genes constitute approximately 30,000 entities, of which approximatively 2% encode proteins. But protein post-translational modifications (PTMs), involving specific chemical alterations, have resulted in the formation of approximately two million protein entities [8]. Previous studies have identified more than 400 different types of PTMs that play crucial roles in numerous cellular functions, including cellular proliferation, metabolism, and signal transduction [9]. PTMs are taking part in various biological processes and closely associated with the pathogenesis of various tumors [10].

Khib was initially identified as a novel post-translational modification (PTM) on histones in HeLa and mouse testis cells by Dai et al. [11]. It is characterized by the addition of a 2-hydroxyisobutyryl group from the donor molecule 2-hydroxyisobutyryl-CoA to its target protein. Subsequent studies have showed the wide distribution of Khib in both prokaryotes and eukaryotes. With scientist’s efforts, the relationship between Khib and several cancer types has been unveiled gradually. Zhang et al. discovered significant alterations in Khib modification levels within the actin cytoskeleton regulatory pathway in oral squamous cell carcinoma (OSCC), highlighting the importance of Khib in OSCC pathogenesis [12]. Furthermore, Yuan et al. found that cell proliferation in liver malignant tumors could be suppressed by inhibiting the Khib modification levels of ENO1K281 [13]. These findings have motivated this study into the potential association between Khib and OACC, which may pave a new way for understanding OACC mechanisms and developing new targeted treatment.

Through a comprehensive investigation of the proteome and post-translational alterations in OACC, we unraveled the potential role of Khib in driving the progression of this malignancy. This study elucidated the influence on the enzyme activity within the glycolysis pathway, shedding light on a plausible mechanistic explanation for OACC advancement.

## Materials and methods

### Sample preparation

Tumor tissues and adjacent normal tissues which were collected from four OACC patients in Shenzhen People’s Hospital, located in Guangdong province of China, were pooled as OACC-T (mixed OACC-tumor tissues) and OACC-N (mixed adjacent normal tissues). The Shenzhen People’s Hospital medical ethics committee gave approval for this study (No. LL-KY-2019173). The participants were provided with an explanation of the research and subsequently signed an informed consent form. After being instantaneously snap-frozen in liquid nitrogen, the tissues were kept at − 80 °C. The analysis of the proteome [14] and quantitative ubiquitylomics of post-translational modifications of ubiquitination [15] in OACC were performed by using these same samples.

### Protein extraction

The samples were transferred from a − 80 °C refrigerator to a liquid nitrogen mortar that had already been precooled. Liquid nitrogen is added in, and the sample is fully ground into powder. With quadruple-volume lysis buffer, each sample was independently lysed and sonicated. The supernatant was transferred to a centrifuge tube, spined at 12,000 × g for 10 min at 4 °C, and a BCA kit was used to determine the protein content.

### Trypsin digestion

For protein digestion, samples were diluted in lysis buffer, and trichloroacetic acid (TCA) was added in. After precipitating at 4 °C for approximately 2 h and centrifuging, the precipitate was washed three times with − 20 °C acetone. Then, the precipitate was dried, and a final concentration of 200-mm triethylammonium bicarbonate (TEAB) was added. Samples were digested overnight by trypsin 1:50 (Trypsin: protein). Liquid was reduced with dithiothreitol (DTT) followed by iodoacetamide (IAA) alkylation. Please see the supporting information for the detailed methods.

### Enrichment of peptides

IP buffer was used to dissolve the peptides. 2-hydroxyisobutyrylated resin was added to the liquid. It was then shaken and incubated at 4 °C overnight. The resin was then cleaned twice with deionized water and four times with IP. The resin-bound peptide sequence was then eluted three times with 0.1% trifluoroacetic acid (TFA), collected, and vacuum-frozen-dried. After that, C18 ZipTips desalted the eluent for LC/MS analysis.

### LC–MS analysis

Solvent A was used to dissolve the digested peptides, and the NanoElute ultra performance liquid chromatography system (UPLC) was used to separate them. The UPLC detailed establishment is shown in as detailed methods of table (Supplementary file 1). The peptides were divided using the UPLC. A capillary ion source was used to ionize them. Tims-TOF Pro mass spectrometry was then used to analyze the data. Please see the supporting information for the detailed methods.

### Database searching

Using MaxQuant (1.6.6.0), secondary mass spectral data were matched to Homo sapiens human (Supplementary Table 1). The reverse decoy database was incorporated to estimate the false-positive rate (FPR) caused by random alignment. Additionally, a shared contamination database was integrated into the database to eliminate protein contamination in the identification outcomes. The detailed methods were shown in Supplementary file 1.

### Bioinformatics methods

#### Differential expression analysis

Mass spectrometry was used to identify each sample’s the protein corresponding signal abundance. The differential expression level (ratio) was detected by label-free quantification (LFQ). DEPs and DHMPs were selected with cutoff conditions of fold change (FC) ≥ 1.2 (log2-fold change ≥ 0.26) or ≤ 1/1.2 (log2-fold change ≤  − 0.26).

#### Subcellular localization

We predicted and described the subcellular localization of the DEPs and DHMPs using WoLF PSORT (v.0.2 http://www.genscript.com/psort/wolf_psort.html).

#### Motif analysis

The 2-hydroxyisobutyrylated protein sequence model, which consists of 10 amino acids upstream and 10 amino acids downstream of the Khib site was analyzed using the Soft MoMo (motif-x algorithm) (v.5.0.2).

#### Functional enrichment

All differentially expressed proteins as well as all differentially 2-hydroxyisobutyrylated modified proteins (DHMPs) database accession were searched against the DAVID Database (https://david.ncifcrf.gov/tools.jsp) for GO category functional classification and KEGG pathway analysis. GO, as we all know, consists of three groups: molecular function, cellular compartment, and biological process. The top 20 *p* values of the most substantially enriched categories from the results of GO category functional categorization and KEGG pathway analysis are displayed in a bubble plot using the OECloud tools (https://cloud.oebiotech.com/task/). Functional categorization or pathway is represented by the bubble plot’s vertical axis, and the enrichment score is represented by the horizontal axis value. The number of proteins is indicated by the size of the bubbles. Circle colors indicate enrichment significance *p* values.

#### Protein–protein interaction network (PPI)

The DHMPs were searched in the STRING database (v.11.5) for PPI. We retrieved all interactions with a confidence score higher than 0.4 (moderate confidence) from STRING (https://cn.string-db.org/). The software Cytoscape was used to display the interactive network from STRING. The software Cytoscape (v.3.8.2) was used to display the PPI from STRING. The top 10 hub proteins of hyper-DHMPs and hypo-DHMPs were selected by cyto-Hubba analysis. The most interconnected molecular clusters in the network were identified by Cytoscape’s MCODE plugin.

## Results

### Clinical characteristics

**Fig. 1:**
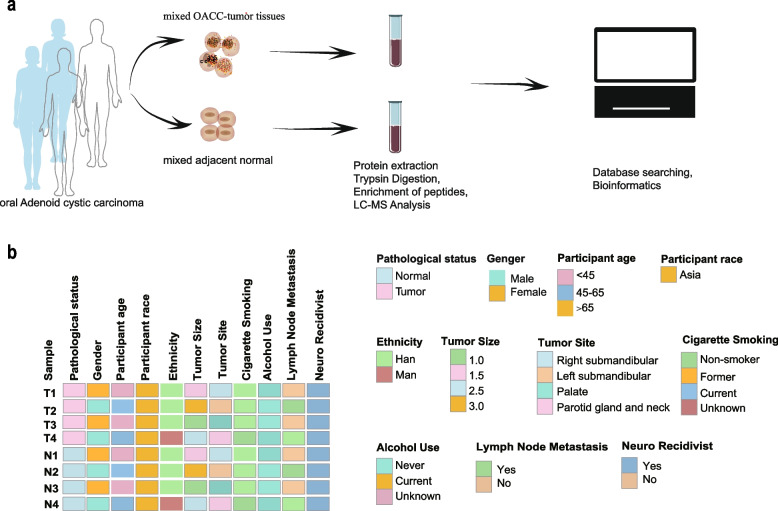
**a** Flow chart. **b** Heatmap of patient clinical characteristics

The flow chart showed the experiment process (Fig. 1a). OACC-tumor samples and the adjacent normal samples were collected from four patients diagnosed with adenoid cystic carcinoma. Subsequently, both group tissues underwent protein extraction, trypsin digestion, peptide enrichment, and LC–MS analysis. To analyze the role of Khib in OACC, a comprehensive bioinformatics analysis was conducted. As shown in Fig. 1b, two female-gender and two male-gender OACC-patients were included in this study. The age of all patients ranged from 23 to 64 years old. The tumor size for all patients ranged from 1.0 to 3.0 cm. All patients were not smoking addict or alcohol addict. Half of the patients had lymph node metastasis. All tumors showed perineural invasion. The detailed clinical data are shown in the Supplementary Table 2.

### Analysis of DEPs in OACC

**Fig. 2:**
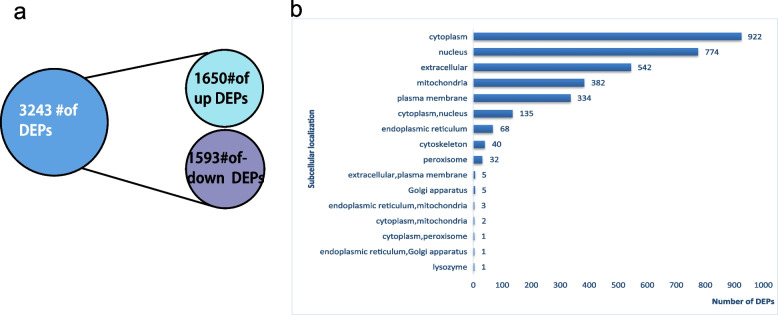
Proteomics screening of DEPs and GO and KEGG functional enrichment bubble plots of DEPs. **a** The quantity of upregulated and downregulated DEPs. **b** Subcellular structure localization and classification of DEPs

There were 5715 identified proteins in all, 4454 of which could be quantified (Supplementary Table 1). The cutoff conditions were fold change (FC) ≥ 1.2 (log2-fold change ≥ 0.26) or ≤ 1/1.2 (log2-fold change ≤  − 0.26). A total of 3243 DEPs were identified, of which 1650 were upregulated and 1593 were downregulated (Fig. 2a). Figure 2b presents the subcellular localization of the DEPs, which revealed that they are primarily distributed in the cytoplasm (922), nucleus (774), and extracellular space (542).

**Fig. 3:**
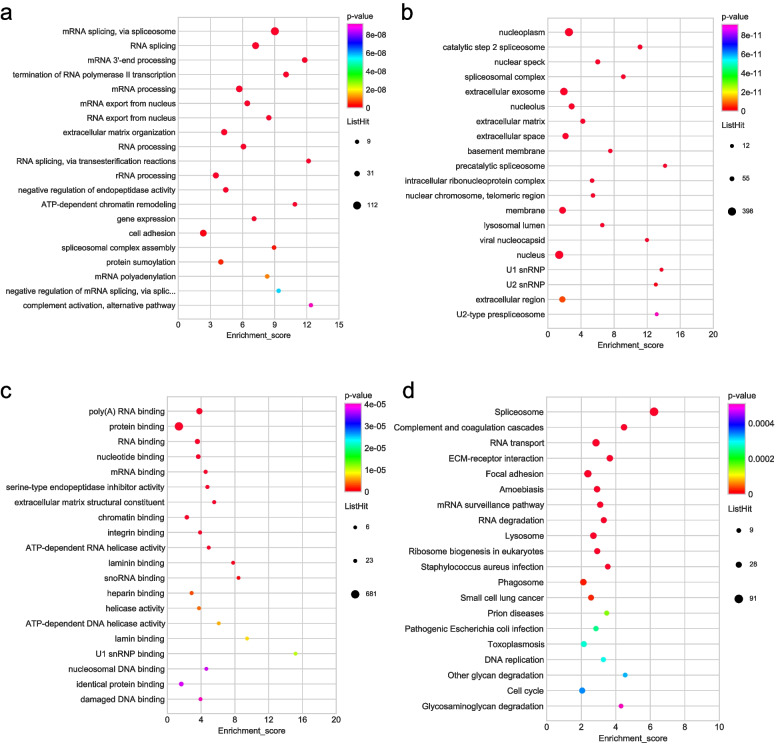
GO and KEGG functional enrichment bubble plots of upregulated DEPs. **a** The GO classifications of upregulated DEPs in the biological processes. **b** The GO classifications of upregulated DEP in the cellular component. **c** The GO classifications of upregulated DEP in the molecular functions. **d** The upregulated DEPs in the KEGG functional enrichment

We evaluated the GO category functional categorization (Supplementary Table 3) and KEGG pathway analysis (Supplementary Table 4) for DEPs in OACC. Figure 3a reveals that mRNA splicing and RNA splicing as well as mRNA export from the nucleus were the three most critical biological process categories for upregulated DEPs. Additionally, nucleoplasm, the extracellular exosome, and catalytic step 2 spliceosome were the three most critical cellular component categories for upregulated DEPs (Fig. 3b). Most of the upregulated DEPs were engaged in not only poly(A) RNA binding but protein binding according to the molecular function classification (Fig. 3c). The top 3 significant KEGG pathways of upregulated DEPs were spliceosome, complement and coagulation cascades, and RNA transport (Fig. 3d).

**Fig. 4:**
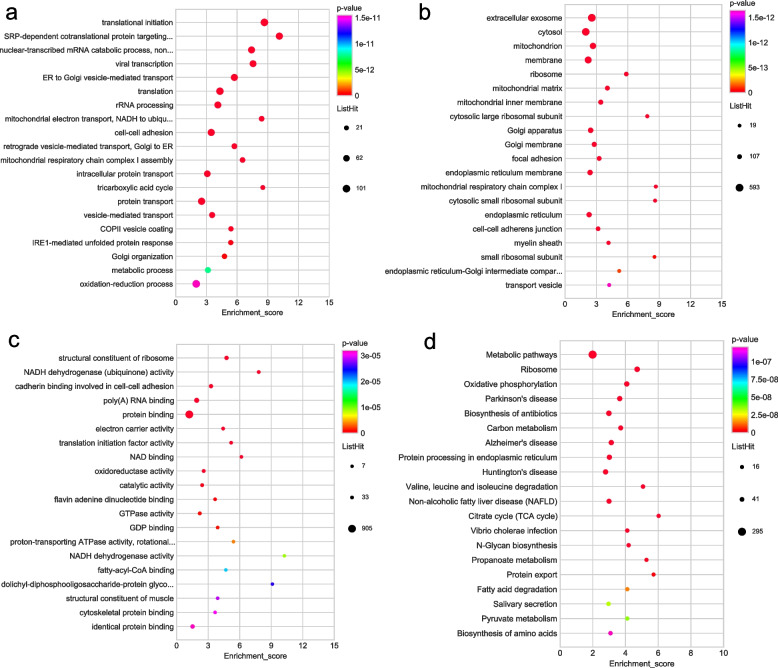
GO and KEGG functional enrichment bubble plots of down-regulated DEPs. **a** The GO classifications of downregulated DEPs in the biological processes. **b** The GO classifications of downregulated DEPs in the cellular component. **c** The GO classifications of downregulated DEPs in the molecular functions. **d** The downregulated DEPs in the KEGG functional enrichment

The majority of the downregulated DEPs were involved in translational initiation as well as SRP-dependent cotranslational protein targeting to the membrane within the biological process categories (Fig. 4a). The majority of DEPs that were downregulated linked to various cellular component category including the extracellular exosome, cytosol and mitochondrion (Fig. 4b). Furthermore, a significant proportion of the downregulated DEPs were found to be associated with molecular functions such as the structural constituent of ribosome, NADH dehydrogenase (ubiquinone) activity (Fig. 4c). The analysis of KEGG pathways revealed that downregulated DEPs were notably enriched in several metabolic pathways, including oxidative phosphorylation and carbon metabolism (Fig. 4d).

### Analysis of differentially 2-hydroxyisobutyrylated modified proteins (DHMPs) in OACC

**Fig. 5:**
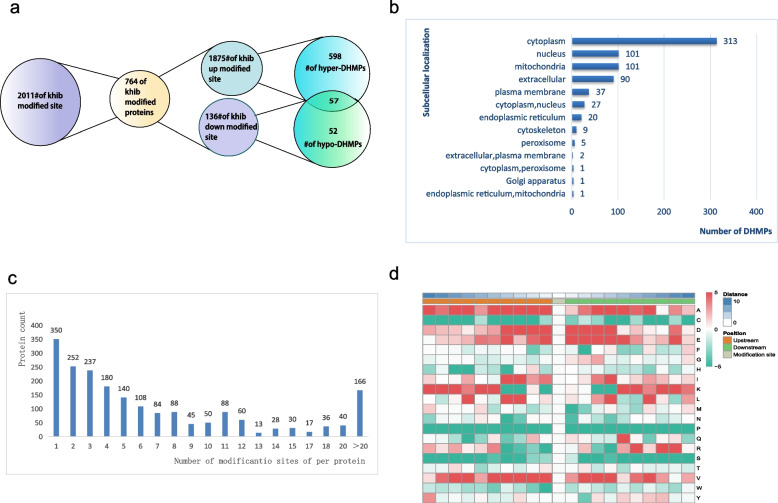
Systematic profiling of DHMPs. **a** Number of DHMPs and differentially Khib modification sites. **b** Subcellular localization and classification of DHMPs. **c** Distribution of Khib in one protein. **d** Motif analysis heatmap of Khib

With the condition of fold change (FC) ≥ 1.2 (log2-fold change ≥ 0.26) or ≤ 1/1.2 (log2-fold change ≤  − 0.26), 2011 Khib sites on 764 proteins (differentially 2-hydroxyisobutyrylated modified proteins, DHMPs) were screened out between OACC-T and OACC-N. A total of 1875 Khib sites for 655 proteins were considered hyper-modified (hyper-DHMPs) and 136 Khib sites for 109 proteins were considered hypo-modified (hypo-DHMPs) (Fig. 5a).

The subcellular localization of DHMPs constituted a considerable portion in the cytoplasm (313), mitochondrion (101), and nucleus (101) (Fig. 5b). More than half of DHMPs have one to four Khib sites (Fig. 5c). Motif analysis heat map of Khib was conducted (Fig. 5d). The color green represents a notable reduction in the abundance of the amino acid near the Khib sites. Conversely, red is indicative of a significant enrichment of the amino acid in this area. Among the amino acids considered, alanine (A) and valine (V), which possess hydrophobic side chain groups, the highest degree of enrichment was observed within positions ranging from − 10 to + 10 positions. Additionally, it was observed that aspartic acid (D) and glutamic acid (E), two acidic amino acids with negatively charged residues, frequently present in these locations. On the other hand, proline (P), cysteine (C), and serine (S) demonstrated the lowest frequency of occurrence in these locations. Consequently, proteins harboring A, D, E, or V at appropriate locations while lacking C, P, or S expected to be more favorable substrates for Khib.

**Fig. 6:**
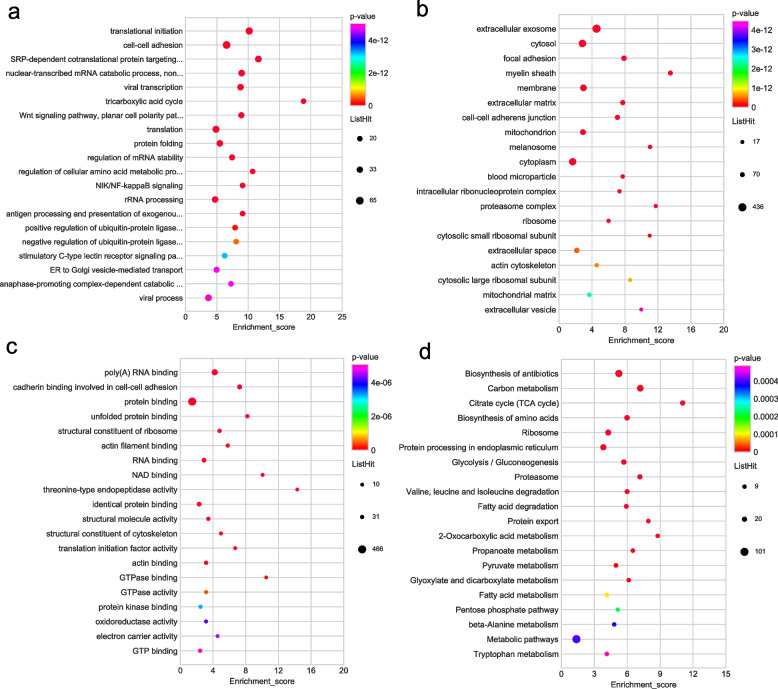
GO and KEGG functional enrichment bubble plots of the hyper-modified DHMPs. **a** The GO classifications of hyper-modified DHMPs in the biological processes. **b** The GO classifications of hyper-modified DHMPs in the cellular component. **c** The GO classifications of hyper-modified DHMPs in the molecular functions. **d** KEGG functional enrichment of hyper-modified DHMPs

To find the significant enrichment property of DHMPs, we performed GO and KEGG pathway enrichment on the basis of hyper-DHMPs and hypo-DHMPs, respectively (Supplementary Tables 5 and 6). According to the GO classification analysis, most of the hyper-DHMPs were involved in translational initiation, and cell–cell adhesion within the biological process categories (Fig. 6a). Within the cellular component category, most of the hyper-DHMPs were involved in the extracellular exosome, cytosol, and focal adhesion (Fig. 6b). Additionally, the hyper-DHMPs were mainly engaged in poly(A) RNA binding, cadherin binding involved in cell–cell adhesion, and protein binding in the MF category (Fig. 6c). We presented the top 20 pathways from DHMPs based on the KEGG pathway analysis. The KEGG pathway analysis of hyper-DHMPs was particularly associated with metabolism such as carbon metabolism, the tricarboxylic acid cycle, and glycolysis/gluconeogenesis (Fig. 6d).

**Fig. 7:**
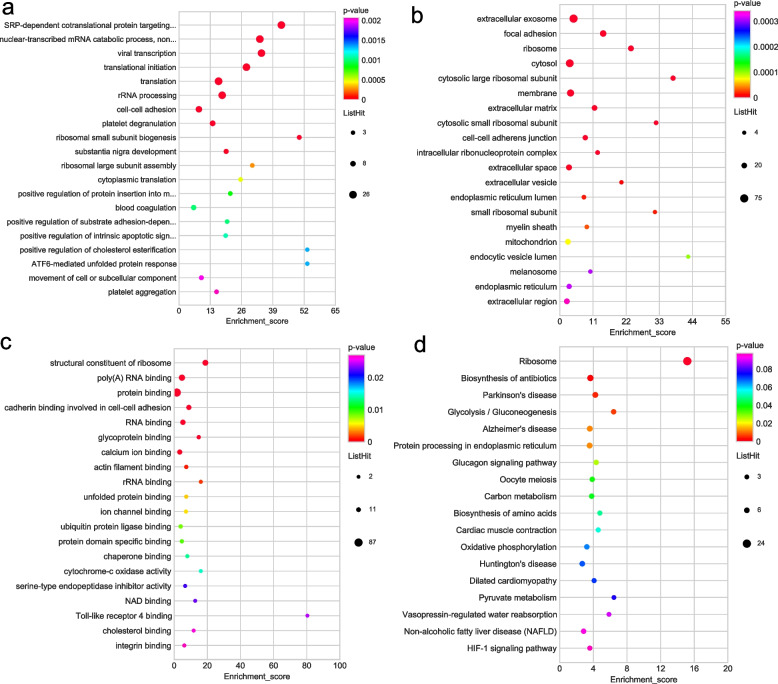
GO and KEGG functional enrichment bubble plots of the hypo-modified DHMPs. **a** The GO classifications of hypo-modified DHMPs in the cellular component category. **b** GO classifications of hypo-modified DHMPs in the biological process category. **c** The GO classifications of hypo-modified DHMPs in and the molecular function category. **d** KEGG functional enrichment of hypo-modified DHMPs

SRP-dependent cotranslational protein targeting to membrane and nuclear-transcribed mRNA catabolic process, non-stop decay were the top two biological process categories (Fig. 7a). Within the category of cellular components, the majority of the hypo-DHMPs were involved in extracellular exosome and focal adhesion (Fig. 7b). The majority of hypo-DHMPs were classified into structural constituent of ribosome, poly(A) RNA binding in the molecular function category (Fig. 7c). The most significant KEGG pathways of hypo-DHMPs were ribosome, biosynthesis of antibiotics, Parkinson’s disease, and glycolysis/gluconeogenesis (Fig. 7d). There was a profound interrelation between the glycolysis pathway and oncogenesis in previous investigations.

### PPI and significant cluster identified by MCODE

**Fig. 8:**
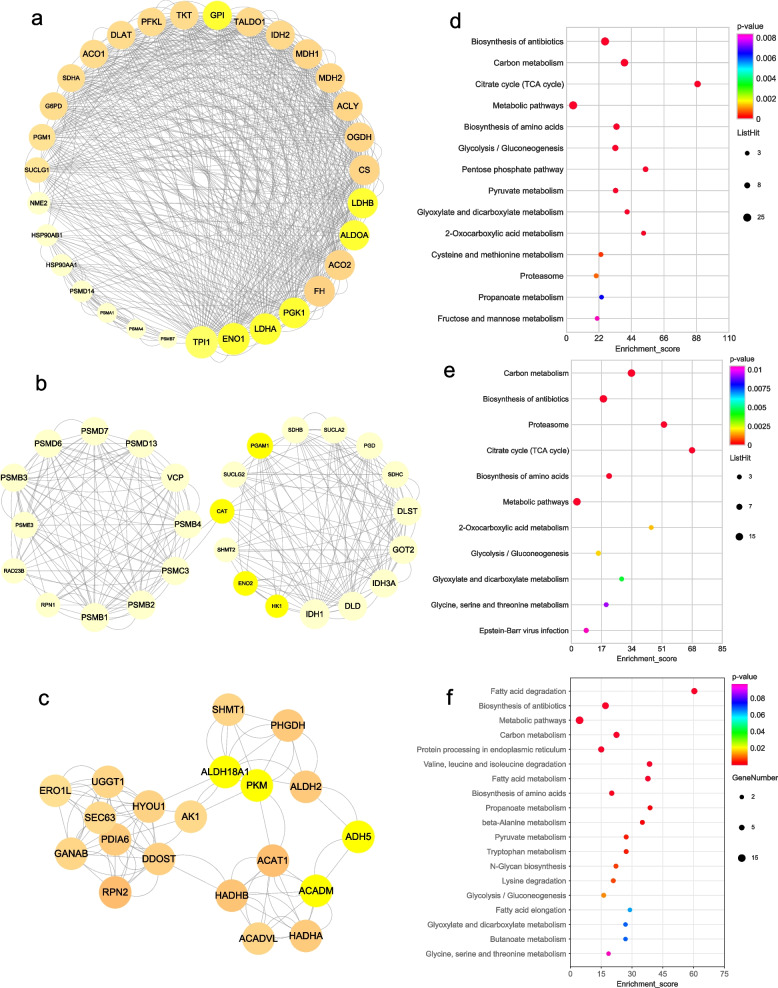
Cluster analysis of hyper-modified DHMPs and KEGG analyses. **a** Protein molecular cluster 2 of hyper-modified DHMPs (score = 19.9). **b** Protein molecular cluster 4 of hyper-modified DHMPs (score = 8.8). **c** Protein molecular cluster 6 of hyper-modified DHMPs (score = 4.9). **d** Bubble plots of cluster 2 KEGG functional enrichment. **e** Bubble plots of cluster 4 KEGG functional enrichment. **f** Bubble plots of cluster 6 KEGG functional enrichment

One hundred seventy-one hyper-modified DHMPs from top ten KEGG pathways of the DHMPs were used to construct the protein connections by the STRING database. Cytoscape (v3.8.2) was used to plot the protein–protein interaction Networks (PPI). The PPI network contained 168 nodes and 6624 edges (Supplementary Fig. 1A). Hyper-modified DHMPs were sorted in accordance with degree of interaction. We identified GAPDH, TPI1, HSPA8, PKM, PGK1, and ENO1 as the top six proteins exhibiting the strongest interactions with other proteins (degree ≥ 144) (Supplementary Table 7). Notably, GAPDH, TPI1, PKM, PGK1, and ENO1 play crucial roles in the glycolysis/gluconeogenesis pathway. Employing the MCODE plugin of Cytoscape, we detected six molecular clusters within the PPI network (score ≥ 4.5) (Supplementary Fig. 1B–G). Specifically, cluster 2 (score = 19.9), clusters 4 (score = 8.8), and clusters 6 (score = 4.9), which contained pivotal proteins of the glycolysis/gluconeogenesis pathway (Fig. 8a-c). Furthermore, KEGG analysis revealed a significant correlation between the proteins in clusters 2, 4, and 6 and glycolysis pathway (Fig. 8d-f, Supplementary Table 8).

**Fig. 9:**
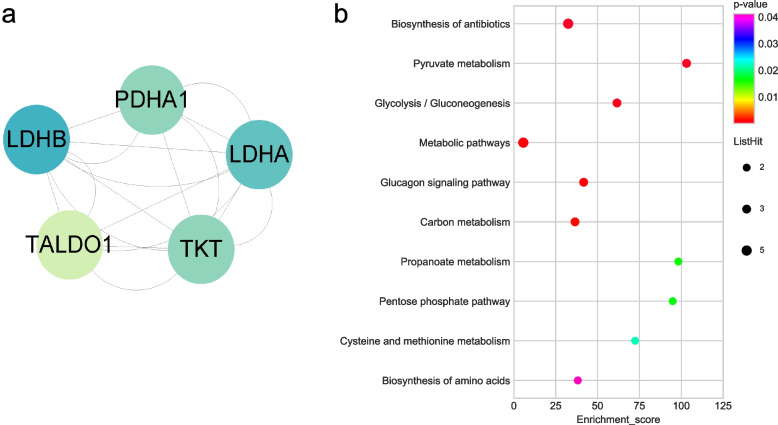
Cluster analysis of hypo-modified DHMPs and KEGG analyses. **a** Protein molecular cluster 2 of hypo-modified DHMPs (score = 4.5). **b** KEGG functional enrichment bubble plots of cluster 2. The yellow color of circles indicates that the DHMPs are related to the glycolysis pathway. The *Y*-axis corresponds to pathway, the X-axis represents the ratio of the differential genes in a specific pathway to all genes in the pathway, the size of the bubble indicates the number of differential genes in the pathway, the bubble color changes from purple-blue-green-red, and the smaller the *p* value is, the greater the significance

The PPI network based on the hypo-modified DHMPs from the top ten KEGG pathways of hypo-DHMPs was constructed on String database and plotted using Cytoscape. The PPI network contained 51 nodes and 926 edges (Supplementary Fig. 2A). GAPDH, SEC61A1, RPS3A, RPL4, RPL9, and RPL3 as the top six proteins exhibiting the strongest interactions with other proteins (Supplementary Table 7). GAPDH which is the center protein of the PPI network (degree = 64) is an essential protein of glycolysis pathway. Employing the MCODE plugin of Cytoscape, we detected two molecular clusters within the PPI network (Supplementary Fig. 2B, C, Fig. 9a). The KEGG enrichment of protein clusters 2 showed that proteins in this cluster were significantly related to glycolysis pathway (Fig. 9b, Supplementary Table 9).

### The Khib of glycolysis pathway enzymes in OACC

**Fig. 10:**
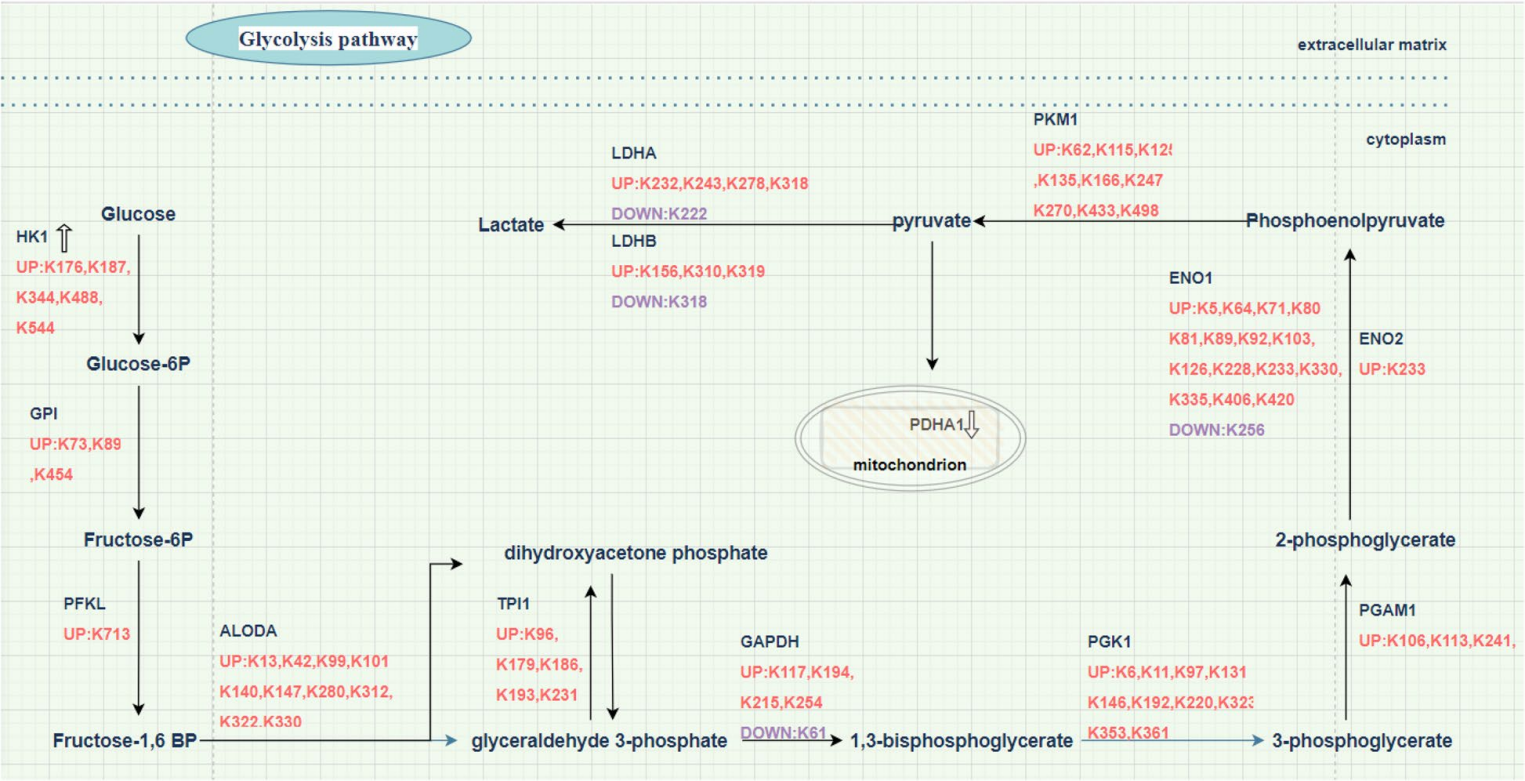
The Khib of glycolysis pathway enzymes. Different colors indicate different regulation. Red indicates that the expression of the Khib modification change is upregulated and purple indicates downregulated. The protein with down arrow indicates that the expression of the protein is downregulated, and up arrow indicates upregulated while proteins without an arrow mean no significant difference between OACC-T and OACC-N

All ten of the crucial glycolysis-related enzymes were modified by Khib (Fig. 10). The hyper Khib-modified sites of HK1 were K176, K187, K344, K488, and K544. The hyper Khib-modified sites of GPI were K73, K89, and K454. Of PFKL, K713 was hyper-modified with Khib. The hyper Khib-modified sites of ALODA were K13, K42, K99, K101, K140, K147, K280, K312, K322, and K330. The hyper Khib-modified sites of TPI1 were K96, K179, K186, K193, and K231. The hyper Khib-modified sites of GAPDH were K117, K194, K215, and K254 while GAPDH was hypo Khib-modified at K61. The hyper Khib-modified sites of PGK1 were K6, K11, K97, K131, K146, K192, K220, K323, K353, and K361. The hyper Khib-modified sites of PGAM1 were K106, K113, and K241.The hyper Khib-modified sites of ENO1 were K5, K64, K71, K80, K81, K89, K92, K103, K126, K228, K233, K330, K335, K406, and K420 while ENO1 was hypo Khib-modified at K256. The Khib of ENO2 was hyper-modified at K233. The hyper Khib-modified sites of PKM1 were K62, K115, K125, K135, K166, K247, K270, K433, and K498. The Khib of LDHA were hyper-modified at K232, K243, K278, and K318 and hypo-modified at K222. The Khib of LDHB were hyper-modified at K156, K310, K319, and hypo-modified at K318. The Supplementary Table 10 showed the detailed fold change of glycolysis enzymes. PADH1 expression level was downregulated while HK1 expression level was upregulated.

## Discussion

Numerous previous studies have demonstrated that Khib participated in diverse biological activities, including glycolysis. Wu et al. conducted a comprehensive analysis of Khib proteome sites in lung cancer cell globally, revealing alterations in ten pivotal glycolysis enzymes due to Khib modification. Among these enzymes, seven experienced substantial modifications with over ten Khib residues [16]. Additionally, Huang et al. determined that the reduction of Khib levels on pivotal glycolysis enzymes significantly impairs their activities. Compared to the control group, hypo Khib-modified cells display notably lower intermediate metabolite concentrations in the glycolysis pathway, indicating decreased enzyme function [17].

Cancer cells’ abnormal energy metabolism may affect several associated metabolic pathways, influencing many biological processes for satisfying their increased growth demands and survival under a range of stress circumstances [18]. Regulation of the glycolysis pathway is one of the “hallmarks of cancer.” Consequently, targeting glycolysis remains attractive for therapeutic intervention. For example, Xu et al. revealed that Chrysin suppressed the glycolysis pathway by reducing HK-2 in tumor tissue, disrupting the energy supply required for tumor growth and inhibiting tumor cell proliferation [19].

With cutoff conditions of fold change (FC) ≥ 1.2 (log2-fold change ≥ 0.26) or ≤ 1/1.2 (log2 fold-change ≤  − 0.26), a total of 2011 Khib sites were identified on 764 proteins. We conducted a systematic bioinformatics analysis for illustrating Khib landscape in OACC. The glycolysis/gluconeogenesis pathway was the most significantly enriched KEGG pathways of both hyper-DHMPs and hypo-DHMPs in this study. In the glycolysis pathway, all 10 pivotal enzymes were modified by Khib (Fig. 10, Supplementary Table 10). Several studies have shown that acetylation is crucial for stability in the glycolytic pathway, while the mechanisms of other PTMs, such as Khib, have rarely been reported.

Hoper-DHMPs and hypo-DHMPs were analyzed by Cytoscape plugin cytoHubba based on stress. This study has identified glyceraldehyde-3-phosphate dehydrogenase (GAPDH) and enolase 1 (ENO1) as prominent constituents among the top 10 proteins (Supplementary Table 7). GAPDH, exchanging glyceraldehyde-3-phosphate (G3P) for 1,3-biphosphoglycerate (1,3-BPG) [20], is essential in aerobic glycolysis of numerous cancers [21–23]. In this study, Khib of GAPDH was hyper-modified at K254 (fold change: 1.29). Chen et al. conducted an analysis of GAPDH acetylation levels and measured GAPDH enzyme activity proposing that hyper-modified acetylation of GAPDH at K254-enhanced GAPDH enzyme activity and tumor cell proliferation [24]. Khib of GAPDH in K254 may play a critical role in the glycolysis pathway to promote cell growth and tumorigenesis. ENO1, a high-energy intermediate, facilitates the conversion of 2-phosphoglycerate to the phosphoenolpyruvate (PEP). ENO1 is intricately linked to the pathogenesis of cancer. Previous study showed that the FAK/PI3K/AKT pathway whose downstream signals could elevate the glycolysis to significantly facilitate non-small cell lung cancer proliferation and metastasis would be activated by upregulated ENO1 [25]. In this study, the Khib in ENO1 showed upregulation trends at 15 sites and K228 was one of the most significant hyper-modified sites (fold change: 3.59). K228 is the key residues that mediate ENO1 catalytic activity [26]. The recent study revealed that lysine residue K228 was on the ENO1 surface, rendering them susceptible to modification by Khib [17]. When a bulky and hydrophilic 2-hydroxyisobutyryl group replaces the positively charged lysine side chain at K228, it may disrupt cofactor binding and induce conformational changes that impact substrate binding and turnover. Consequently, the activity of ENO1 is enhanced in response to the heightened glucose consumption associated with cancer malignancy [13, 17]. Based on this premise, we hypothesized that K228hib also elevated glycolysis in OACC cells by augmenting the activity of ENO. The previous study showed that acetylation of ENO at lysine residue 257 can neutralize its positive charge and change binding site geometry, thereby disturbing the electrostatic binding potential and impairing the enzyme’s ability to bind substrates, ultimately inhibiting the glycolysis pathway [27, 28]. In this study, a noteworthy observation was made regarding the sole and significant downregulation of K256hib in ENO1. This residue is located in close proximity to K257 and may possess a similar functionality to K257. K256hib potentially exerted an inhibitory effect on the binding capacity of ENO1 by inducing alterations in the shape of the binding site and the electrostatic binding potential. We postulated that upregulation of K228hib and downregulation of K256hib both increased the catalytic efficiency of ENO1 to promote glycolysis pathway which favor OACC progression.

Phosphoglycerate kinase 1 (PGK1) is one of the top ten hub proteins of hoper-DHMPs based on Cytoscape cyto-Hubba analysis (Supplementary Table 7). PGK1 which catalyzes the reversible transfer of a phosphoryl group from 1, 3-bisphosphoglycerate (1, 3-BPG) to ADP and produces 3-phosphoglycerate (3-PG) and ATP [29] is composed of N- and C-terminal [30]. The N-terminal domain of PGK1 allowed 1,3-BPG or 3-PG to bind, and the C-domain allowed ADP to bind [31]. We found that all Khib sites of PGK1 are upregulation trends in OACC-tumor tissues, including the K323 site (fold change: 1.33). According to previous studies, acetylation at K323, located in its C-terminal domain of PGK1, enhanced its catalytic efficiency by increasing its affinity for ADP/ATP [32] and promoted the proliferation, glycolysis, and tumorigenesis of liver cancer [33]. Thus, we supposed that hyper Khib-modified of PGK1 at the K323 site may also enhance proliferation and tumorigenesis of OACC by enhancing the binding affinity of ADP/ATP to promote PGK1 activity and glycolysis.

Hexokinase (HK), the first rate-limiting enzyme in the glycolysis pathway, plays a critical role in cancers. In instances where tumor cells experience a severe glucose deficit, HK1 assumes a more prominent role in facilitating glycolytic reactions compared to HK2, owing to its lower Km. The expression of HK1 has been shown upregulated trend which is beneficial to cancer cell proliferation by enhancing glycolysis in some cancer types, including pancreatic malignant tumor [34], bladder carcinoma [35], and ovarian malignant tumor [36]. In this study, compared with OACC-N, the expression of HK1 is upregulated in OACC-T. This upregulation serves to promote glycolysis, thereby enhancing oral proliferation and migration of OACC. It is worth noting that all Khib of HK1 are upregulation trends in OACC-tumor tissues, especially at K488 (fold change:5.39) and K187 (fold change: 2.43). Zhang et al. reported that phosphorylation of Y732 probably improved the catalytic efficiency of HK1 by causing the homodimerization of the enzyme to break down, which raised the enzyme’s affinity for the substrate glucose [37]. To date, a limited number of studies contain data on the relationship between Khib of HK1 and its enzyme activity, necessitating further investigation in this domain. We supposed that hyper Khib-modified of HK1 probably also caused the homodimers to separate, thereby increasing catalytic efficiency of HK1 and enhancing glycolysis, ultimately facilitating tumorigenesis and metastasis. Pyruvate dehydrogenase A1 (PDHA1) belongs to the enzyme complex known as pyruvate dehydrogenase complex, which acts a gate-keeper enzyme between the mitochondrial citric acid cycle and glycolysis. Pyruvate dehydrogenase is crucial for the cancer metabolism and its suppression in cancer cells can boost the Warburg effect. Previous studies have shown a significant downregulation of PDHA1 expression in breast cancer [38], ovarian carcinoma [39], and gastric cancer [40] resulting in enhanced glycolysis and an association with poor prognosis. However, the correlation between the PDHA1 protein expression and the metabolism and biological behavior of OACC is unclear. In this study, PDHA1 was underwent significant down trend expression (fold change: 0.76), which suggest that the metabolic change in OACC cells to rely more on glycolysis may be due to attenuated mitochondrial function through inhibition of PDHA1. In conclusion, targeting Khib may be an attractive OACC therapy.

## Conclusion

We investigated the DEPs and DHMPs between OACC-T and OACC-N using LC–MS/MS-based quantitative proteomics and bioinformatics analysis. Khib played an important role in potential mechanism of OACC by affecting enzyme activity of glycolysis pathway.

### Supplementary Information

**Additional file 1: Supplementary text.** Detailed research methods. **Supplementary Fig. 1.** PPI and cluster of hyper-modified DHMPs. **Supplementary Fig. 2.** PPI and cluster of hypo-modified DHMPs. **Supplementary Table 1.** Statistics of Mass Spectrometry Data. **Supplementary Table 2.** Clinical characteristics of OACC patients. **Supplementary Table 3.** Go enrichment analyses of differentially expressed proteins. **Supplementary Table 4.** KEGG pathway enrichment of differentially expressed proteins. **Supplementary Table 5.** Go enrichment analyses of differentially expressed and 2-hydroxyisobutyrylated modified proteins. **Supplementary Table 6.** KEGG pathway enrichment of differentially expressed and 2-hydroxyisobutyrylated modified proteins. **Supplementary Table 7.** Top 10 hub proteins in hyper-and hypo modified DHMPs based on degree. **Supplementary Table 8.** KEGG enrichment in up cluster 1–6. **Supplementary Table 9.** KEGG enrichment in down cluster 1–2. **Supplementary Table 10.** The Khib of glycolysis pathway enzyme.

## Data Availability

The datasets generated during and/or analyzed during the current study are available from the corresponding author on reasonable request.

## Abbreviations

ACC: Adenoid cystic carcinoma
OACC: Oral ACC
OACC-T: OACC-tumor samples
OACC-N: Adjacent normal samples
Khib: Lysine 2-hydroxyisobutyrylation
PTM: Post-translational modification
DEPs: Differentially expressed proteins
DHMPs: Differentially expressed and 2-hydroxyisobutyrylated modified proteins
MS: Mass spectrometry
UPLC: Performance liquid chromatography
FDR: False discovery rate
GO: Gene Ontology
BP: Biological process
CC: Cellular component
MF: Molecular function
KEGG: Kyoto Encyclopedia of Genes and Genomes
PPI: Protein-protein interaction
MCODE: Molecular complex detection
GAPDH: Glyceraldehyde-3-phosphate dehydrogenase
ENO1: Enolase 1
PGK1: Phosphoglycerate Kinase 1
PKM: Pyruvate Kinase M1
HK1: Hexokinase 1
PDHA1: Pyruvate dehydrogenase A1
