# Supplementary material for: Analysis of proteome and post-translational modifications of 2-hydroxyisobutyrylation reveals the glycolysis pathway in oral adenoid cystic carcinoma
Source: World J Surg Oncol. 2023 Sep 23;21:301. doi: 10.1186/s12957-023-03155-x (PMC10517466; doi:10.1186/s12957-023-03155-x)
Supplement: Supplementary file 1 — Additional file 1: Supplementary text. Detailed research methods. Supplementary Fig. 1. PPI and cluster of hyper-modified DHMPs. Supplementary Fig. 2. PPI and cluster of hypo-modified DHMPs. Supplementary Table 1. Statistics of Mass Spectrometry Data. Supplementary Table 2. Clinical characteristics of OACC patients. Supplementary Table 3. Go enrichment analyses of differentially expressed proteins. Supplementary Table 4. KEGG pathway enrichment of differentially expressed proteins. Supplementary Table 5. Go enrichment analyses of differentially expressed and 2-hydroxyisobutyrylated modified proteins. Supplementary Table 6. KEGG pathway enrichment of differentially expressed and 2-hydroxyisobutyrylated modified proteins. Supplementary Table 7. Top 10 hub proteins in hyper-and hypo modified DHMPs based on degree. Supplementary Table 8. KEGG enrichment in up cluster 1–6. Supplementary Table 9. KEGG enrichment in down cluster 1–2. Supplementary Table 10. The Khib of glycolysis pathway enzyme. [file 12957_2023_3155_MOESM1_ESM.zip › Detailed research methods and Supplementary figure.pdf]

# Analysis of proteome and post-translational modifications of 2-hydroxyisobutyrylation reveals the Glycolysis pathway in oral Adenoid cystic carcinoma

Sining Chen[1,2#], Dandan Li[1#], Zhipeng Zeng[1], Wei Zhang[1], Hongliang Xie[3], Jianming Tang[3], Shengyou Liao[1], Wanxia Cai[1], Fanna Liu[2]*, Donge Tang[1]*, Yong Dai[1,4,5]*

[#] These authors contributed equally to this study.

1. Clinical Medical Research Center, The Second Clinical Medical College of Jinan University (Shenzhen People's Hospital), Shenzhen, Guangdong 518020, China.
2. Institute of Nephrology and Blood Purification, The First Affiliated Hospital of Jinan University, Jinan University, Guangzhou 510632, China
3. Department of Oral and Maxillofacial Surgery, Stomatological Medical Center, The Second Clinical Medical College of Jinan University (Shenzhen People's Hospital), Shenzhen, Guangdong 518020, China.
4. Taizhou Research Institute, Southern University of Science and Technology, Taizhou 318000, China
5. Guangxi Key Laboratory of Metabolic Disease Research, 924st Hospital, Guilin, 541002, China;

## Detailed research methods

## Protein extraction

The sample was grinded by liquid nitrogen into cell powder in a liquid nitrogen pre-cooled mortar. And four- volumes of lysis buffer (1% Triton X-100, 1% protease inhibitor, 50 μm PR-619, 3 μm TSA, 50 mm NAM) was added in the sample. After sonication three times on ice and centrifugation in 12000×g for 10 min at 4 ℃, the supernatant was collected and the protein concentration in the supernatant was determined by BCA kit.

## Trypsin digestion

Equal amount of protein from OACC tumor samples (OACC-T) and the adjacent normal samples (OACC-N) were taken to enzymatic digestion. And lysis buffer was added to adjust the total volume equally. Trichloroacetic acid(TCA) (20%w/v) was slowly added in it. After precipitating at 4°C about 2 hour and centrifuging in 4500×g for 5 min, the precipitate was washed with pre-cooled acetone three-times, dried, and dissolved by 200 mm triethylammonium bicarbonate (TEAB). Trypsin was added on a scale of 1:50(Trypsin: protein) for overnight. After added with dithiothreitol(DTT) at 56°C about 30 min to reduce, samples were added with iodoacetamide (IAA) and put at room temperature for 15 min in dark to alkylate.

## Enrichment of peptides modified by 2-hydroxyisobutyrylation

The peptides were dissolved in IP (100 mM NaCl, 1 mM EDTA, 50 mM Tris-HCl, 0.5% NP-40, pH 8.0), and the supernatant was transferred to 2-hydroxyisobutyrylated Resin (antibody resin product number (PTM-804), from Hangzhou Jingjie Biotechnology Co., Ltd., PTM Bio), placed on a rotary shaker at 4 °C, gently shaken and incubated overnight. Then resin was washed 4 times with IP and twice with deionized water. Finally, 0.1% trifluoroacetic acid (TCA) was used to elute the resin-bound peptide sequence for three-times, and the eluent was collected and vacuum-freeze-dried. Then the eluent was desalted by C18 ZipTips to LC/MS analysis.

## LC-MS Analysis

The digested peptides were dissolved by liquid chromatography mobile phase A (containing 0.1% formic acid and 2% acetonitrile) and separated by NanoElute ultra performance liquid chromatography system. The gradient of mobile phase B (containing 0.1% formic acid and 100% acetonitrile) was set as follows: 6–22%: 0–43 min; 22–30%: 43–56 min; 30–80%: 56–58 min; 80%: 58–60 min, all at a constant flow rate of 450.00 nl/min(table 1). The peptides were separated by Ultra Performance Liquid Chromatography (UPLC), injected into Capillary Ion Source whose voltage was 2.0 kV and analyzed by Tims-TOF Pro MS. The peptide parent ion and its secondary fragments were detected and analyzed by high-resolution TOF. The scanning range of secondary MS is set to 100–1700. One MS scan followed by 10 MS/MS scans in PASEF mode, and the dynamic exclusion time of tandem MS scanning was set to 30 s in order to avoid repeating scanning of the precursor ion.

| time（min） | SolventA（%） | SolventB（%） | flow rate（nl/min） |
|---|---|---|---|
| 0 | 94 | 6 | 450 |
| 70 | 76 | 24 | 450 |
| 84 | 65 | 35 | 450 |
| 87 | 20 | 80 | 450 |
| 90 | 20 | 80 | 450 |
| A: 2% acetonitrile and 0.1% formic acid | | | |
| B: 100% acetonitrile and 0.1% formic acid | | | |

**table 1 The UPLC detailed establishment**

## Database searching

The MaxQuant search engine v1.6.6.0 was used to process the secondary MS data. The proteins were detected in Homo sapiens 9606 (20366 sequences). Tandem mass spectra were searched against human uniprot database concatenated with reverse decoy database. False-positive discovery rate(FDR) was adjusted to < 1%.Trypsin/P was specified as cleavage enzyme allowing up to 2 missing cleavages. the minimum length of the peptide is set to 7 amino acid residues and the maximum number of peptide modifications is set to 5. The mass tolerance for precursor ions was set as 20.0

ppm in First search and 20 ppm in Main search, and the mass tolerance for fragment ions was set as 20.0 ppm. Carbamidomethyl on Cys was specified as fixed modification and acetylation modification and oxidation on Met were specified as variable modifications.

## Supplementary figure

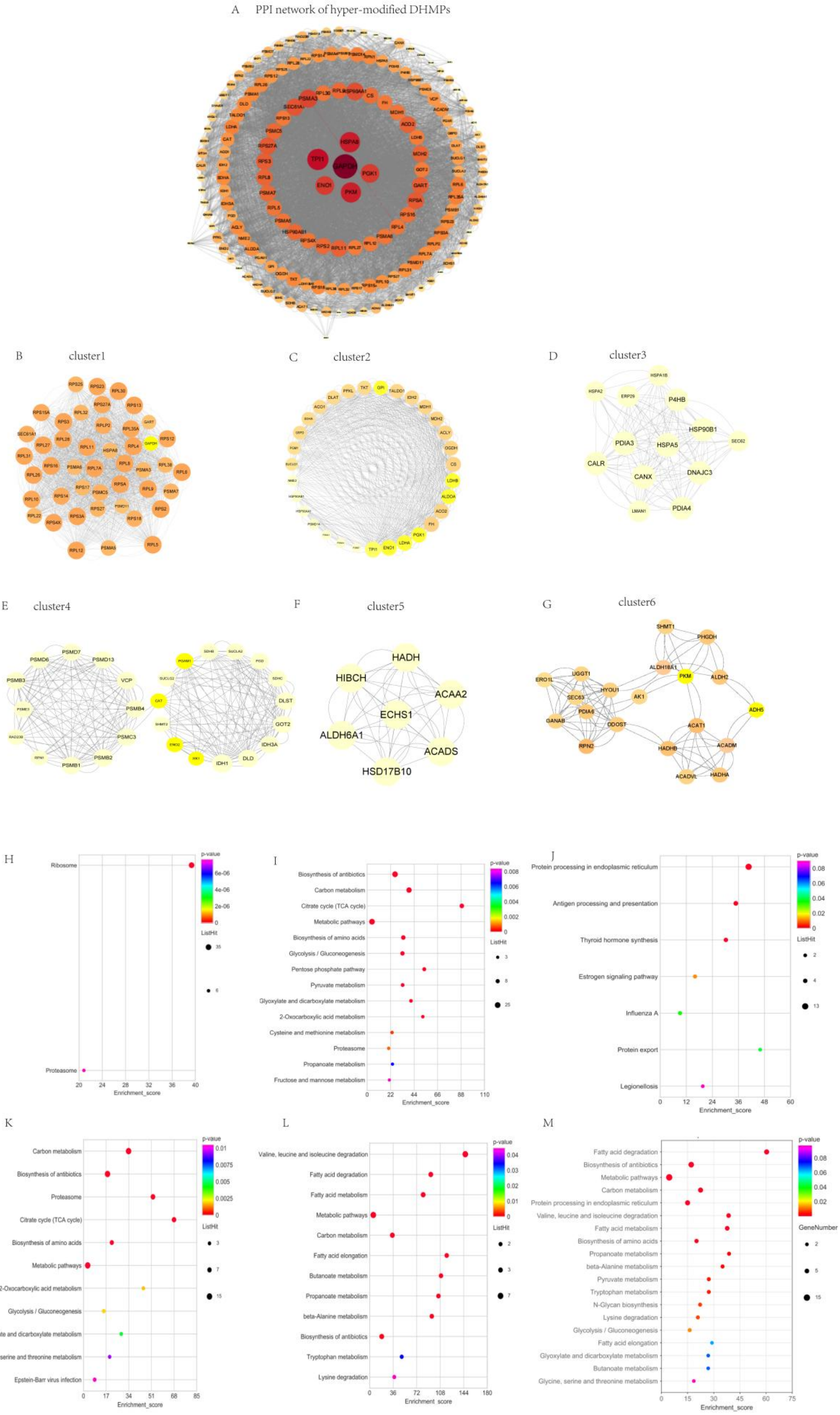


**Supplementary figure1 :PPI and cluster of hyper-modified DHMPs**

(A) the PPI network of 171 hyper-modified DHMPs. (B-G) cluster (1-6) of hyper-modified DHMPs (Score≥4.5). (H-M) Bubble plots of cluster1-6 KEGG functional enrichment.

In PPI network, the color depth of the ellipse represents the degree, the greater the degree, the darker the color. The larger the circle indicates that the more protein it interacts with, the more important that protein is in the network. In bubble plots, the Y axis are pathway, the X axis represents the ratio of the differential genes in a specific pathway to all genes in the pathway. The size of the bubble indicates the number of differential genes in the pathway. The bubble color changes from purple-blue-green-red, and the smaller the p-value, the greater the significance.

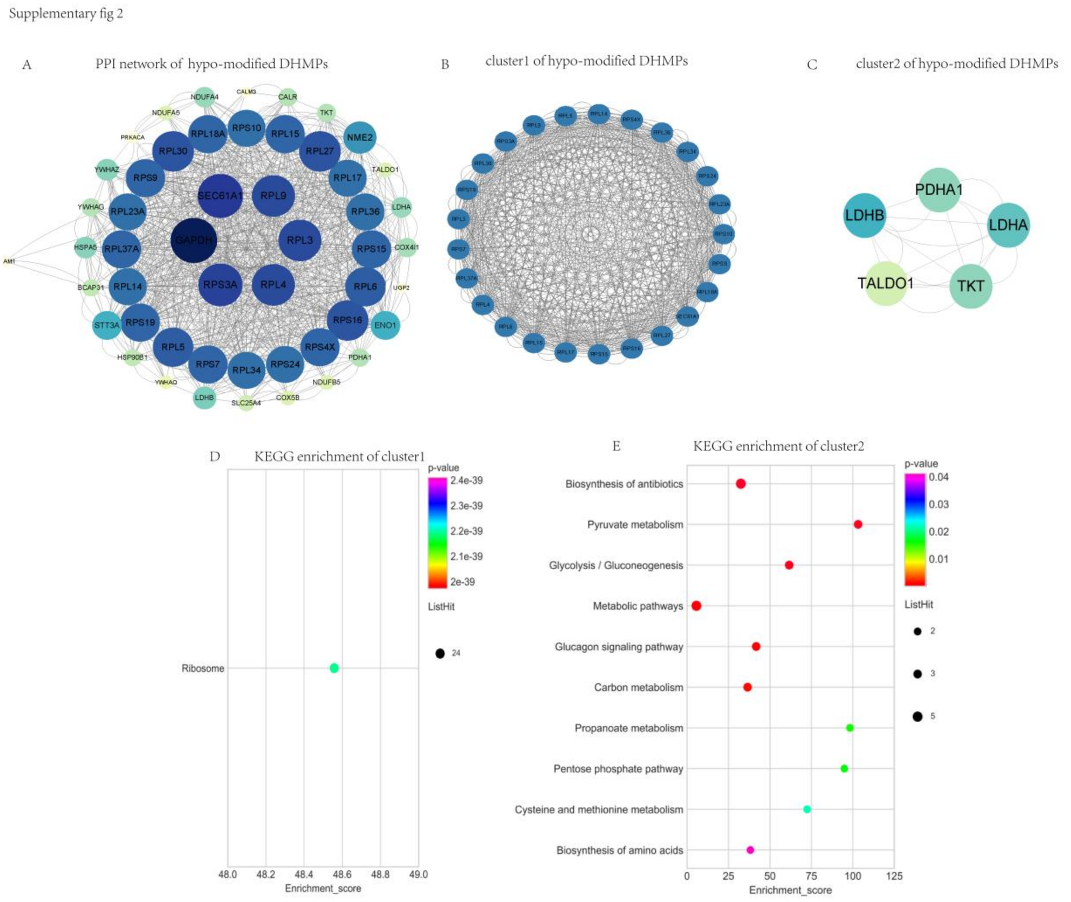


## Supplementary fig2: PPI and cluster of hypo-modified DHMPs

(A) the PPI network of 53 hypo-modified DHMPs. (B)Protein molecular cluster1 of hypo-modified DHMPs (Score≥4.5). (C) cluster2 of hypo-modified DHMPs (Score≥4.5). (D) KEGG functional enrichment bubble plots of cluster1. (E) KEGG functional enrichment bubble plots of cluster2.
